# Perceptions of Attitude, Ethics, and Communication (AETCOM) Modules Among Indian Medical Graduates in Their First Professional Year: An Educational Observational Study

**DOI:** 10.7759/cureus.64611

**Published:** 2024-07-15

**Authors:** Rekha Udgiri, Praveen Ganganahalli

**Affiliations:** 1 Community Medicine, Shri B M Patil Medical College Hospital and Research Centre, BLDE (Deemed to be University), Vijayapura, IND

**Keywords:** cbme, medical students, perception, feedback, aetcom module

## Abstract

Background

The National Medical Commission (NMC) of India has redesigned the graduate medical education program to equip Indian medical graduates with essential information, skills, attitudes, values, and responsiveness as physicians in their initial interactions with the community. Central to this initiative is the Attitude, Ethics, and Communication (AETCOM) module, designed as a guide for educators and institutions to implement a comprehensive, long-term program. This aims to ensure that students develop competency as clinicians, leaders, team players, communicators, lifelong learners, and professionals.

Objectives

The aim of this study is to evaluate students’ perceptions of the AETCOM modules during their first year.

Methodology

This cross-sectional study was conducted at BLDE (Deemed to be University), Vijayapura, India, utilizing self-administered, semi-structured questionnaires for data collection. The study included second- and third-year medical students, with all respondents who submitted their responses being included in the study. The total sample size comprised 123 students.

Results

Ninety-eight percent of the students agreed that the NMC had taken excellent initiative with the AETCOM module and found its duration sufficient. They suggested that the teaching-learning techniques should include more interactive sessions.

Conclusions

Feedback from the AETCOM module is crucial for enhancing its effectiveness, and it should be gathered from all medical colleges to propose necessary improvements.

## Introduction

The National Medical Commission (NMC) of India established a redesigned graduate medical education program to help Indian medical graduates acquire the necessary information, skills, attitudes, values, and responsiveness as primary care physicians. Students must be competent as communicators, clinicians, leaders, healthcare team members, and lifelong learners to achieve the aim [1,2].

The current medical education system prioritizes knowledge acquisition, yet successful health professionals must not only possess knowledge but also practical skills. While students may excel in theoretical knowledge, they often struggle to apply it effectively in practical settings [3,4].

To enable educators and institutions to conduct a long-term program to help students learn the necessary competency domains, the Attitude, Ethics, and Communication (AETCOM) module has been designed as a manual. Finding out what students thought about AETCOM modules was the goal of the current study [5,6].

The AETCOM module is a long-term program incorporated nationwide into the new curriculum to assist students in gaining the knowledge and abilities they need in communication, ethics, and attitudes. A total of 29 components need to be completed between the first and fourth professional years. There are optional and required core competencies for each component. Fifty-four overall competencies need to be obtained, comprising 39 core and 15 optional conative domain competencies [7].

## Materials and methods

An educational observational (cross-sectional) study was conducted at BLDE (Deemed to be University), Vijayapura, India. The study was carried out with student-informed verbal consent and institutional ethics approval in September 2021.

The NMC created AETCOM modules for each year of the MBBS course, then deployed them nationwide for Indian medical graduates in the 2019-2020 cohort. The AETCOM module has been prepared as a guide to facilitate institutions and faculty in implementing a longitudinal program that will help students acquire the necessary competence in the attitudinal, ethical, and communication domains. Accordingly, teaching-learning activities related to ethics and communication are incorporated into the regular teaching program. During the first year of the medical course, five modules will be covered in 34 hours using various teaching, learning, and assessment techniques. The modules are structured as follows: “What does it mean to be a doctor?,” “What does it mean to be a patient?,” “The doctor-patient relationship,” “The Foundations of Communication - 1,” and “The Cadaver as our first teacher.”

Study population

All subjects were second-year medical undergraduates who finished their first year.

Inclusion criteria

All second-year medical course students were included in the study.

Exclusion criteria

The students who were not present and unwilling to participate were excluded from the study.

Sampling technique

A convenience sampling technique was used to enroll the students in the study.

Study method

A self-administered semi-structured questionnaire was used to gather feedback from undergraduate students in first-year modules (five modules), and the questionnaire’s validity was pretested. To validate the questionnaire and determine the sample size, a pilot study involving 26 students was conducted. A 5-point Likert scale (ranging from 1 to 5) was used to assess the perception of structured questions like the appropriateness of the module, competencies and objectives, the teaching-learning methods, duration allotted, satisfactory implementation, and assessment methods. Open-ended questions were also asked regarding the disadvantages of the module, suggestive measures, recommendations for implementation, and overall reflection. Perceptions about each module were gathered using Google Forms.

## Results

Among the 123 students who participated in the study, 68 (55%) were females and 55 (45%) were males. The participants’ average age group was 20.41 ± 0.886. Table 1 displays the information collected in median and interquartile ranges for a 5-point Likert scale for all five courses. According to this, the mean and median values obtained for each module were either acceptable or good/very good on the Likert scale, lying in the 50th and 75th percentiles, respectively.

**Table 1:** Median and interquartile ranges for a 5-point Likert scale across all five AETCOM modules AETCOM, Attitude, Ethics, and Communication

		Module I	Module II	Module III	Module IV	Module V
Mean		3.49	3.44	3.44	3.41	3.37
Median		4	3	3	3	3
Standard deviation		0.549	0.575	0.498	0.557	0.578
Quartiles	25	3	3	3	3	3
	50	4	3	3	3	3
	75	4	4	4	4	4

**Figure 1:**
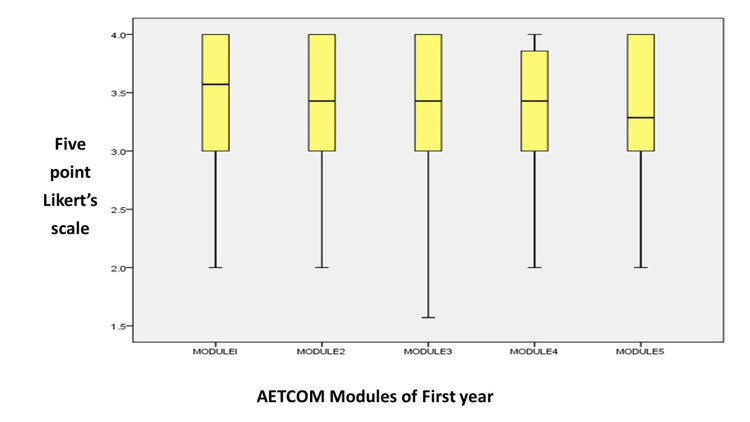
Box plot representation of all five modules using a 5-point Likert scale AETCOM, Attitude, Ethics, and Communication

Eighty-nine percent of the students said the time allocated for each lesson was adequate. Of all the modules, the maximum number of students (65%) thought that the module on the doctor-patient relationship, module 1.3, was the most helpful, followed by modules 1.1 and 1.2, in that order. Of them, only 10% thought all the modules were helpful (Figure 1). The figure explains the median values obtained by the Likert scale given by the students. The line in the middle of the box represents the data’s median (the 50th percentile), and the box’s right side represents the data’s third quartile (the 75th percentile).

All pupils were given open-ended questions, and repetition was avoided while composing their answers. According to 98% of the students, the AETCOM module is mandatory for Indian medical graduates. Students gave several reasons for this, like the worthy initiative by NMC, which helps understand the relation between health and disease, the importance of communication, respect for cadavers, etc. Their understanding of attitudes and ethics in the medical field was aided by it. They also believed that to be successful in their work, doctors needed to possess strong moral principles and effective communication abilities. It also aided in instilling the profession’s values and culture in them and comprehending the function of physicians in the modern world. Their understanding of patient behavior and interaction improved thanks to the modules, which will help them become better clinicians.

About implementing all five modules in the first professional year, their responses were as follows: It helped them understand how to behave and the mindset of patients and doctors. They are happy that it is being implemented. It was necessary to learn ethics regarding the hospital setting. They said that awareness of proper AETCOM skills with patients play an important role in the success of medical professionals. As they had clinical postings in the second year, modules helped them to implement communication skills between themselves and the patient. It helps to a great extent for history taking, as they experience all these in their clinical postings.

Related to teaching-learning methods adopted for the AETCOM module, the responses they quoted are as follows: It was an interactive session; teaching was excellent, good, and appropriate. Had a lot of fun experiences and ended up understanding and learning a lot. They suggested showing more video demonstrations.

Regarding the faculty’s involvement in an activity, they said that all departments equally took part in helping the students. They conducted it properly. It was excellent and good. The faculty members were very cooperative and explained the concepts nicely. They said it was a good effort made by all faculty members.

Most respondents said that the assessment method implemented for AETCOM was appropriate, good, and well assessed by writing reflection. Some suggested conducting quiz competitions, multiple-choice questions, and viva.

Overall reflections about the AETCOM module by the students are as follows: It gave them insight into the medical profession and made them realize the immense responsibility and perseverance that comes with it; it helped in dealing with patients and allowed them to learn the problems and solutions of the ethical problem; they got to learn how to empathize with the patients; and they learned the skills and precautions needed while handling a patient. Throughout their professional lifetimes, doctors encounter human misery. A comprehensive comprehension of patients’ illnesses, their perceptions of suffering, coping strategies, and the physician’s role is necessary for a well-rounded approach to the patient care experience. Modules helped them show good leadership qualities and be great team members; they should obey orders, and sometimes vice versa. It helped them provide their best services to society and learn empathy and sympathy.

The disadvantages of the modules included students finding the duration too time-consuming and the theory classes boring.

Regarding all five modules that aided them in their second professional year, all students provided positive responses, with only 2% expressing minimal benefit.

Suggestions for improving the modules included various inputs: Should we increase interactive sessions and reduce the allotted time? It would be beneficial to incorporate real-life cases to enhance engagement. Some participants felt no further improvements were necessary, noting the modules were already implemented effectively, but recommended adding time management skills.

Their recommendation for future implementation of these modules was to continue them, as they are deemed essential for the medical profession.

## Discussion

A new graduate medical education curriculum has been developed by the NMC of India to help Indian medical graduates acquire the necessary knowledge, skills, attitudes, values, and responsiveness as primary community physicians. The AETCOM module has been developed as a manual to assist educators and institutions in putting into practice a long-term curriculum that will help students gain the skills they need in the communication, ethics, and attitude domains. One of the most important aspects of competency-based education is assessment. As a result, student input was gathered to offer recommendations for advancement. There is currently very little literature for comments on these modules following the NMC’s adoption of the AETCOM module [1].

In the present study, 98% said the AETCOM module is required for Indian medical graduates. Similar findings were observed by Bidikar et al. [8], where most students (98%) opined that the AETCOM module had improved their future interactions with patients. They are also valued positively for gaining insight into patients’ moralities and being useful for future practice. This shows that implementing the AETCOM module has helped the undergraduate student learn AETCOM skills.

The present study’s average Likert scale score regarding all five AETCOM modules was good. Vijayasree [9] also found that 84% of students said it was satisfactory, followed by 1% just satisfactory, and 4% highly satisfied. This shows that AETCOM modules will help them acquire competencies as a longitudinal program.

In reflection writing, our study observed that the AETCOM module helped them in their clinical posting in the second year and also helped them to implement communication skills between them and patients. Similarly, observation was found in a study conducted by Srabani and Sundaram [10] in their study, which highlighted that the AETCOM module helped the students to a great extent with history taking, as they experienced all of these in their clinical postings. This gives importance to teaching the AETCOM module, which benefits the students.

The present study suggested more interactive sessions and reduced the time allotted. Srabani and Sundaram [10] observed the same student response. Therefore, they suggested more small-group discussion teaching methods.

The maximum number of students (65%) said module 1.3 (doctor-patient relationship) was very useful among all the modules in another study conducted by Varma et al. [11], wherein the majority (74%) of the students considered communication skills training important for undergraduate medical students, and 68.4% felt they would like to have communication skills training as part of their curriculum. Another study also observed the same response from the students [12].

Shilpa et al. [13] highlighted that most students have realized the importance of empathy for building a good doctor-patient relationship and its role in effective patient management. Overall, the AETCOM module integrates essential components addressing ethics, professionalism, and communication. This equips Indian medical graduates to effectively serve as primary points of contact with the community.

Mallika et al. [14] and Sharma and Mahajan [15] concluded in their studies that students have realized the importance of empathy for building a good doctor-patient relationship and its role in effective patient management through the AETCOM modules, and good communication skills should be taught and practiced in the medical curriculum to increase clinical competence.

The strongest aspect of the AETCOM module teaching is that undergraduates know the importance of the right attitude and good communication for patients’ healthcare. In contrast, the weakest aspects are proper implementation, assessment, and feedback in spite of the module.

The study is limited to being conducted at a single medical college and is not generalizable to the entire population of undergraduate students. This can be corrected by conducting a multicenter study.

## Conclusions

Based on the analysis, we can say that the NMC of India chose to put the AETCOM module into use, which is not just mandatory, but the skills that each course lists, such as communication, ethics, and attitude, are good and desperately needed for Indian medical graduates. Encouraging students to utilize AETCOM modules systematically during their medical education would assist them in becoming committed, competent medical professionals that the public can trust. The current study recommends modifying a few teaching-learning practices to generate more engaged classes. Every medical institution is required to submit feedback regularly to make suggestions for enhancing the AETCOM courses. A competency-based curriculum should be implemented in every medical facility in place of traditional teaching techniques.

## Appendices

Questionaries for each module (1.1 to 1.5) were recorded separately on a 5-point Likert scale.

**Table 2:** Perception assessment form for first-year undergraduate students regarding the AETCOM module AETCOM, Attitude, Ethics, and Communication

Questions	1 = Very poor	2 = Poor	3 = Acceptable	4 = Good	5 = Very good
The given module is appropriate for the first year.					
The competencies and objectives addressed are clear and feasible.					
The teaching-learning methods adopted were appropriate and feasible.					
The total duration allotted for the module is sufficient.					
The implementation of the module is satisfactory.					
The assessment method conducted is appropriate.					
The module helped you in the second year.					

Open-ended questions: Disadvantages of the modules, suggestive measures for improvement of modules, recommendations for implementing these modules in the future, and overall reflections about the AETCOM module.
